# UHPLC-IM-Q-ToFMS analysis of maradolipids, found exclusively in *Caenorhabditis elegans* dauer larvae

**DOI:** 10.1007/s00216-021-03172-3

**Published:** 2021-02-11

**Authors:** Michael Witting, Ulrike Schmidt, Hans-Joachim Knölker

**Affiliations:** 1 Research Unit Analytical BioGeoChemistry, Helmholtz Zentrum München, Ingolstädter Landstraße 1, 85764 Neuherberg, Germany; 2 Metabolomics and Proteomics Core, Helmholtz Zentrum München, Ingolstädter Landstraße 1, 85764 Neuherberg, Germany; 3 Chair of Analytical Food Chemistry, TUM School of Life Sciences, Technical University of Munich, Maximus-von-Imhof-Forum 2, 85354 Freising, Germany; 4 Faculty of Chemistry, Technische Universität Dresden, Bergstraße 66, 01069 Dresden, Germany

**Keywords:** Ion mobility spectrometry, Lipidomics, Lipid identification, *Caenorhabditis elegans*

## Abstract

Lipid identification is one of the current bottlenecks in lipidomics and lipid profiling, especially for novel lipid classes, and requires multidimensional data for correct annotation. We used the combination of chromatographic and ion mobility separation together with data-independent acquisition (DIA) of tandem mass spectrometric data for the analysis of lipids in the biomedical model organism *Caenorhabditis elegans*. *C. elegans* reacts to harsh environmental conditions by interrupting its normal life cycle and entering an alternative developmental stage called dauer stage. Dauer larvae show distinct changes in metabolism and morphology to survive unfavorable environmental conditions and are able to survive for a long time without feeding. Only at this developmental stage, dauer larvae produce a specific class of glycolipids called maradolipids. We performed an analysis of maradolipids using ultrahigh performance liquid chromatography-ion mobility spectrometry-quadrupole-time of flight-mass spectrometry (UHPLC-IM-Q-ToFMS) using drift tube ion mobility to showcase how the integration of retention times, collisional cross sections, and DIA fragmentation data can be used for lipid identification. The obtained results show that combination of UHPLC and IM separation together with DIA represents a valuable tool for initial lipid identification. Using this analytical tool, a total of 45 marado- and lysomaradolipids have been putatively identified and 10 confirmed by authentic standards directly from *C. elegans* dauer larvae lipid extracts without the further need for further purification of glycolipids. Furthermore, we putatively identified two isomers of a lysomaradolipid not known so far.

**Supplementary Information:**

The online version contains supplementary material available at 10.1007/s00216-021-03172-3.

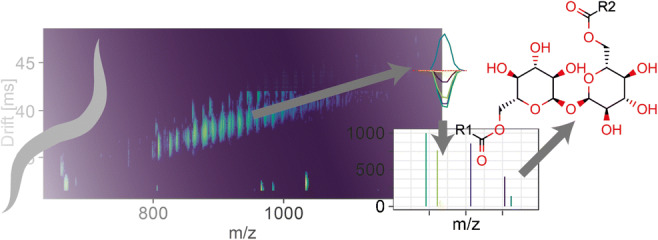

## Introduction

Lipidomics has become an important tool in biomedical research and aims to detect, identify, and ideally quantify all lipids in a given sample [1]. Different lipidomics workflows exist either using direct infusion mass spectrometry (MS) or liquid chromatography (LC) coupled to MS. While the direct infusion approach, also call shotgun lipidomics, is ideal for quantification of lipid species, LC-MS is used in discovery or profiling workflows. Two separation modes are mainly employed: while hydrophilic liquid interaction chromatography (HILIC) separates lipids according to their class, reversed-phase (RP) separates lipid species according to their hydrophobicity. The latter allows a more detailed description of lipid species and their composition. Another emerging tool for lipid analysis is the ion mobility separation (IM). Separation in IMS is based on the differential traveling of ions in a drift gas along an electric field. The velocity of ions is based on their molecular shape, which is expressed as rotational averaged collision cross section (CCS). CCS values help to add further confidence in lipid identification. The combination of IM with MS and tandem MS (IM-MS) is therefore gaining interest [2–5]. In contrast to other parameters, CCS values can be predicted de novo or based on machine learning approaches [6, 7].

Lipids are normally identified based on their characteristic fragmentation pattern. While these patterns are well established for several lipid classes, for new lipids, they have to be determined. Typically, lipid profiling using RP-LC data-dependent acquisition (DDA) is used. However, the stochastic nature of precursor selection and user-set inclusion thresholds lead to a limited coverage, often selecting only well-known, highly abundant lipids. Data-independent acquisition (DIA) represents an interesting alternative. However, different other problems arise from the chimeric fragmentation spectra that are obtained using this acquisition mode. IM as additional separation dimension can help to clean up chimeric spectra. A recent investigation showed that DIA in combination with IM was able to annotate more metabolites in human plasma [8].

The small nematode *Caenorhabditis elegans* (*C. elegans*) is one of the premier model organisms in biomedical research. *C. elegans* normally develops from the fertilized egg through four larval stages into reproductive adults. In order to react to changing environments, organisms *C. elegans* can interrupt its normal life cycle and enter an alternative developmental stage called dauer stage (“dauer”, German for enduring). As compared with normal larvae, dauer larvae show distinct changes in metabolism and morphology to survive unfavorable environmental conditions. Dauer larvae are able to survive for a long time without feeding. Once conditions ameliorate, they develop into normal adults without compromises in lifespan or fertility. *C. elegans* harbors a complex metabolome and lipidome with several different lipid classes, including lipids specific to *C. elegans* [9].

Changes in metabolism enable improved usage of energy resources and include the rerouting of several metabolic pathways [10]. One interesting aspect of dauer larvae is the production of specific glycolipids distinct from glucosylceramides. They have been named maradolipids and are found exclusively in the dauer stage of *C. elegans*. Chemically, they are defined as 6,6′-diacyltrehaloses and have been identified for the first time by Penkov et al. They performed an extraction and purification of glycolipids followed by shotgun analysis of the obtained lipids. Maradolipids contain a high amount of branched chain fatty acids, mostly C15:0iso (> 20 mol%) [11]. An additional study identified lysomaradolipids, containing only a single acyl group [12].

So far, maradolipids have been only analyzed by shotgun lipidomics. However, LC-MS-based workflows are often used for lipid profiling and allow the separation and detection of new lipids and lipid classes [13].

This investigation presents the use of UHPLC and IM in combination with DIA for the analysis of maradolipids. Based on maradolipid standards [15] analyzed to determine their chromatographic and ion mobility behavior as well as fragmentation in positive and negative ionization mode using DIA, we established a workflow for identification of further maradolipids directly from *C. elegans* dauer larvae lipid extract without further purification of the glycolipid fraction. Based on RT, CCS, and DIA fragmentation data, different maradolipids could be putatively identified. In total, 33 maradolipids could be putatively identified (Metabolomics Standard Initiative (MSI) Level 2 [14]), and then, 10 of them were confirmed by an authentic standards (MSI Level 1). Additionally, a 12 lysomaradolipids has been putatively identified, including potential two isomers of LysoMar (17:0). The obtained results show how RT, CCS, and DIA can help in the identification of novel lipids.

## Material and methods

### Chemicals

**Fig. 1:**
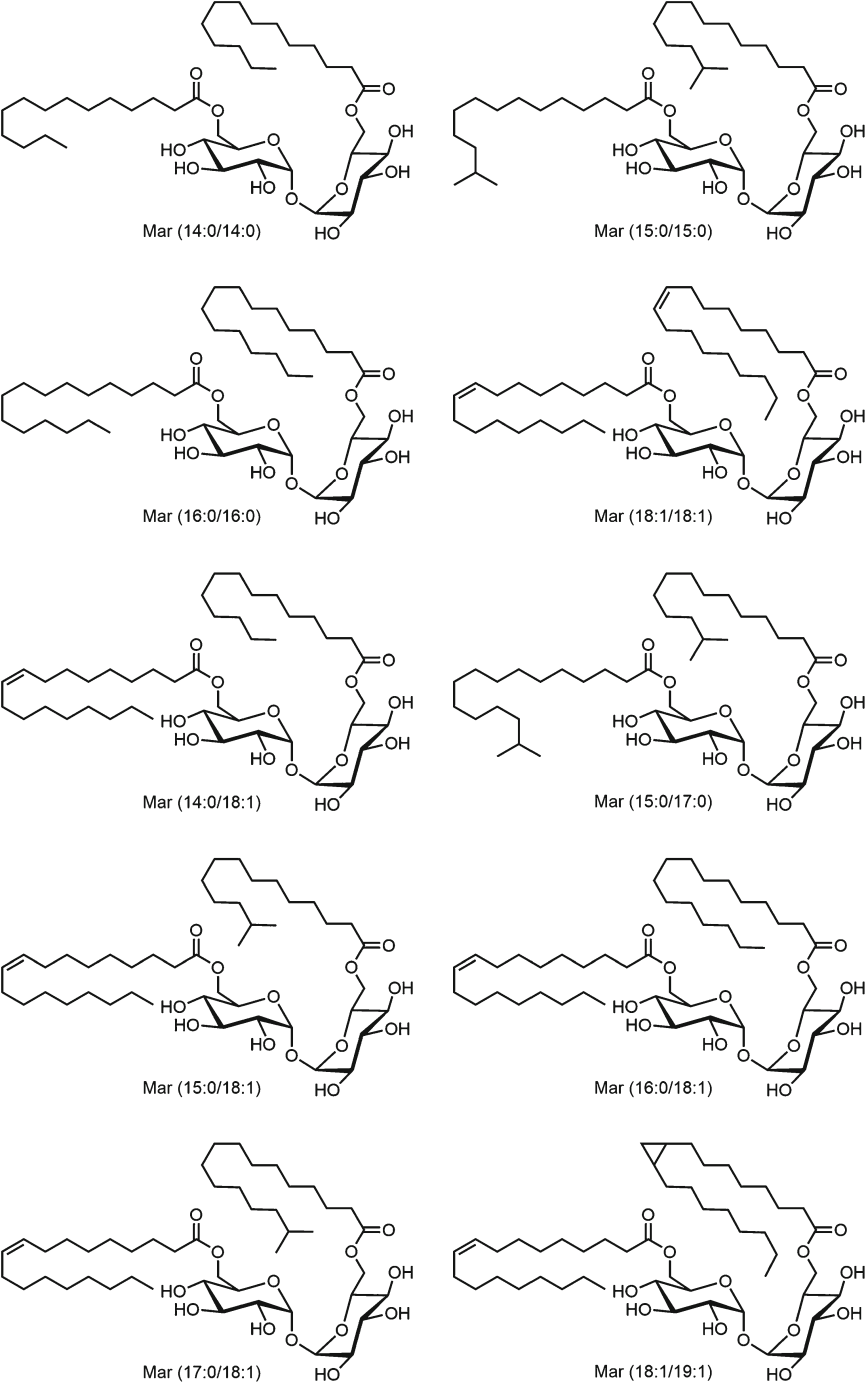
Structure of maradolipid standards synthesized and used in this study

Maradolipid standards have been synthesized using a previously reported procedure [15]. A mix standard consisting of 6-*O*-myristoyl-6′-*O*-myristoyltrehalose (Mar(14:0/14:0)), 6-*O*-(13-methylmyristoyl)-6′-*O*-(13-methylmyristoyl)trehalose (Mar(15:0/15:0)), 6-*O*-myristoyl-6′-*O*-oleoyltrehalose (Mar(14:0/18:1)), 6-*O*-palmitoyl-6′-*O*-palmitoyltrehalose (Mar(16:0/16:0)), 6-*O*-(13-methylmyristoyl)-6′-*O*-(15-methylpalmitoyl)trehalose (Mar(15:0/17:0)), 6-*O*-(13-methylmyristoyl)-6′-*O*-oleoyltrehalose (Mar(15:0/18:1)), 6-*O*-palmitoyl-6′-*O*-oleoyltrehalose (Mar(16:0/18:1)), 6-*O*-(15-methylpalmitoyl)-6′-*O*-oleoylterhalose (Mar(17:0/18:1)), 6-*O*-oleoyl-6′-*O*-oleyoltrehalose (Mar(18:1/18:1)), and 6-*O*-oleoyl-6′-*O*-((2-octylcyclopropyl)octanoyl)trehalose (Mar(18:1/19:1)) was dissolved in methanol (structures are summarized in Fig. 1). All solvents and additives were obtained from Merck/Sigma-Aldrich and were of LC-MS grade (Sigma-Aldrich, Taufkirchen, Germany). Agilent Low Concentration Tune Mix was obtained from Agilent Technologies (Agilent Technologies, Waldbronn, Germany). LC-MS grade water was obtained by purification using a Millipore Integral 3 purification system yielding 18.2 MΩ and a TOC < 5 ppb (Merck Millipore, Darmstadt, Germany).

### *C. elegans* cultivation and extraction

*daf-2(e1370) *Mutants were obtained from the Caenorhabditis Genetics Center (CGC) and grown under standard conditions according to Brenner et al. on Nematode Growth Medium (NGM) [16]. To obtain dauer larvae, synchronized L1 larvae were obtained by bleaching and seeded onto NGM plates and grown at 25°C. Once sufficient amounts of dauer larvae were obtained, worms were washed off the plates using an M9 buffer and washed three times. Lipids were extracted according to Bligh and Dyer [17]. The chloroform phase was evaporated to dryness and redissolved in 60% iPrOH/35% ACN/5% H_2_O (*v*/*v*/*v*) prior to analysis.

### Stepped field analysis of maradolipid standards

Collisional cross sections (CCS) of maradolipid standards were collected using the stepped field method by Stow et al. using an Agilent 6560 DT-IM-Q-ToFMS equipped with a Dual Agilent Jet Stream ESI source (Agilent Technologies, Waldbronn, Germany) [18]. Ionization source parameters were as follows: (+) mode: Vcap 4000 V, nozzle voltage 2000 V, fragmentor 400 V, gas temperature 250°C, gas flow 12 L/min, nebulizer 40 psig, sheath gas temperature 320 °C, sheath gas flow 11 L/min; (−) mode: Vcap 5500 V, nozzle voltage 2000 V, fragmentor 400 V, gas temperature 250°C, gas flow 12 L/min, nebulizer 40 psig, sheath gas temperature 320 °C, and sheath gas flow 11 L/min. The instrument was operated with N_2_ as drift gas at a pressure of 3.95 Torr. The maradolipid standard mix was diluted in a 50/50 mixture of eluent A and eluent B (see below) and infused using a syringe pump with a flow rate of 500 μL/min. Data was analyzed using the Agilent MassHunter Workstation IM-MS Browser 10.0. IM data has been referenced using either the [M+H]^+^ adduct of Hexakis(1H,1H,3H-perfluoropropoxy)phosphazene (*m*/*z* 922.009799, ^DT^CCS_N2_ 243.64 Å^2^) or the [M+TFA-H]^−^ adduct of Hexakis(1H,1H,3H-perfluoropropoxy)phosphazene (*m*/*z* 1033.987012, ^DT^CCS_N2_ 255.34 Å^2^) from the reference mass solution in positive and negative ionization mode, respectively.

### UHPLC-IM-Q-ToFMS analysis

Chromatographic separation was performed as described by Witting et al. [19]. Lipids were separated using an Agilent 1290 Infinity II UHPLC (Agilent Technologies, Waldbronn, Germany) equipped with Waters CORTECS UPLC C18 column (150 mm × 2.1 mm ID, 1.7 μm particle size) (Waters, Eschborn, Germany). Separation was achieved by a linear gradient from 68% eluent A (40% H_2_O/60% ACN, 10 mM ammonium formate, and 0.1% formic acid) to 97% eluent B (10% ACN/90% iPrOH, 10 mM ammonium formate, and 0.1% formic acid). Mass spectrometry detection was performed using an Agilent 6560 DT-IM-Q-TOF-MS equipped with a Dual Agilent Jet Stream ESI source (Agilent Technologies, Waldbronn, Germany). Ion source parameters were the same as for the stepped field analysis. Ion mobility separation was performed under a single-field conditions with DIA fragmentation using an alternating scheme, switching between low and high collision energy using either 10, 20, or 40 eV. In order to obtain ^DT^CCS_N2_ values, calibration of the IM dimension was performed using the Agilent Low Concentration Tune Mix infused prior to running the sample sequence. Data was preprocessed using the PNNL PreProcessor v2020.03.13 (https://omics.pnl.gov/software/pnnl-preprocessor) with a smoothing in RT direction using 3 data points and in drift direction using 5 data points. Additional saturation repair has been performed [20].

Non-targeted four-dimensional peak picking has been performed using the Agilent MassHunter Workstation Mass Profiler 10.0 software. Minimum peak intensity was set at 100 counts and common organic formula without halogens was used as isotope model. Alignment parameters were as follows: RT tolerance ± 10% + 0.5 min, DT tolerance ± 1.5%, and mass tolerance ± 15 ppm + 2.0 mDa. Calculation of Kendrick mass defects (KMD) and referenced Kendrick mass defects (RKMD) and all further data handling were performed in Microsoft Excel. KMDs and RKMDs were calculated according to equations 1, 2, and 3. A KMD of 0.6094 calculated from the mass of Mar(32:0) was used for the calculation of the RKMDs.

1 $$ \mathrm{KM}=\mathrm{exact}\ \mathrm{mass}\times \frac{14}{14.015650} $$

2 $$ \mathrm{KMD}=\mathrm{KM}-\mathrm{nominal}\ \mathrm{KM} $$

3 $$ \mathrm{RKMD}=\frac{\left(\mathrm{experimental}\ \mathrm{KMD}-\mathrm{reference}\ \mathrm{KMD}\right)}{0.013399} $$

DIA fragmentation data was examined using the Agilent MassHunter Workstation IM-MS Browser 10.0. Mass spectra in the respective drift region of the intact precursor were extracted and checked for fitting fragments. For all fragment candidates, extracted ion chromatograms for the *m*/*z* and the specific drift region were created and compared against the extracted ion chromatogram of the precursor. Fragments with a Pearson correlation coefficient > 0.9 were retained as correct. Width of the EIC window was 0.05 Da, while drift time windows were about 2 ms. Correlation analysis of EICs was performed in R using the correlate function from the XCMS 3.0 package (https://github.com/sneumann/xcms).

## Results and discussion

### Determination of reference ^DT^CCS_N2_ values and RTs

In order to characterize the IM separation of maradolipids, ^DT^CCS_N2_ values of authentic reference standards were determined. Maradolipid standards were infused in a 50/50 mixture of eluent A and B of the later employed chromatographic method. In positive ion mode, maradolipids are ionizing as [M+NH_4_]^+^ adducts during direct infusion as well as [M+FA-H]^−^ adducts in negative mode. This is in agreement with Penkov et al., who detected acetate adducts of maradolipids in negative ion mode. Although [M+Na]^+^ adducts were detected during chromatographic analysis, they were not detected in the direct infusion experiments.

**Fig. 2:**
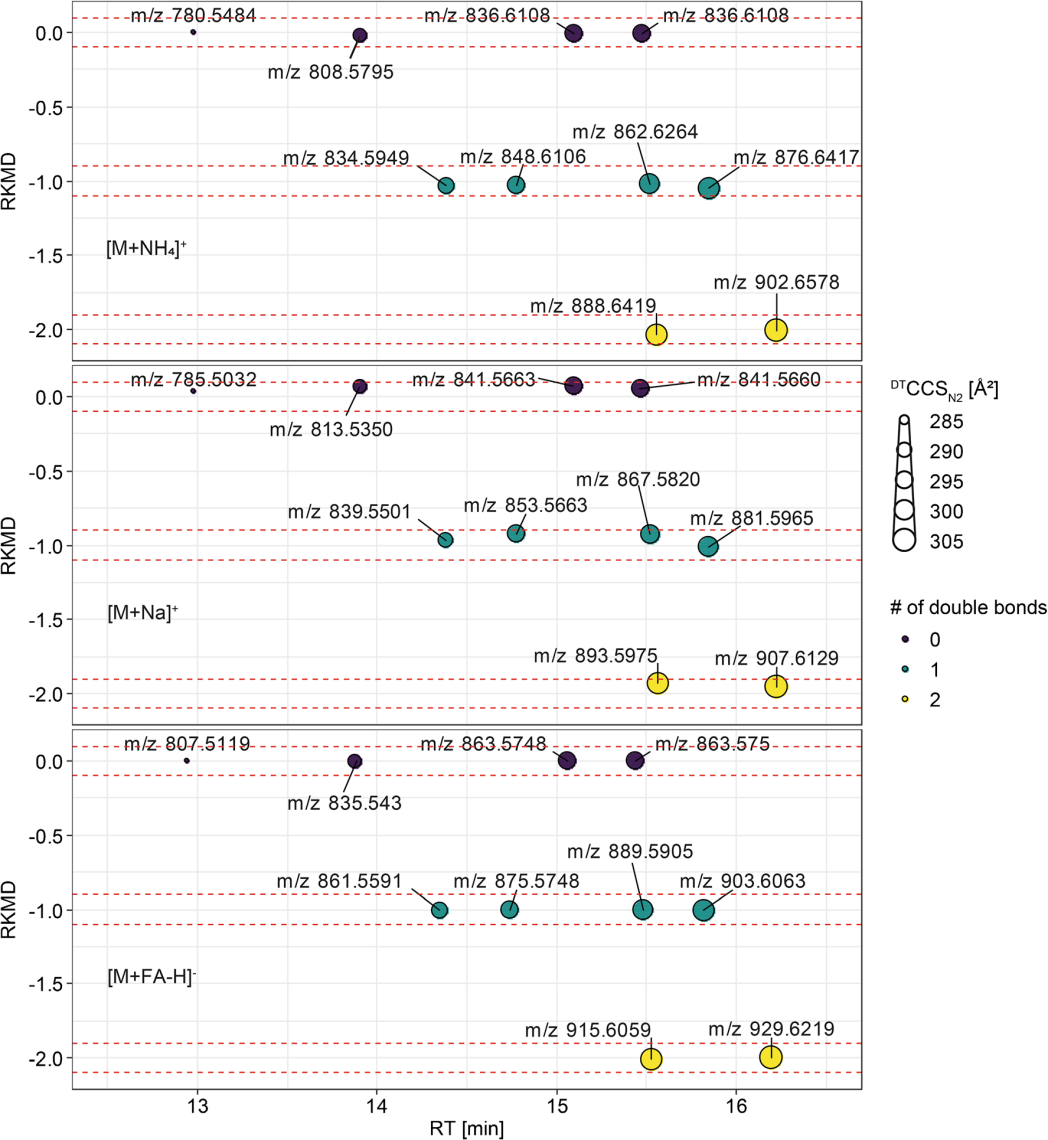
Plots of the RKMD against the RT show horizontal trendlines that can be used for identification of maradolipids. The ^DT^CCS_N2_ value is shown as size of the point. Increasing chain length leads to larger molecular structures hence higher ^DT^CCS_N2_ values and an increased RT on the used RP separation. Different degrees of unsaturation are seen as parallel lines

^DT^CCS_N2_ values of the maradolipid standards were determined using the stepped field method according to Stow et al. [18]. Consistent with other lipid classes, increasing chain length led to increased ^DT^CCS_N2_. In the next step, UHPLC-IM-QToFMS was performed using a single field drift tube experiment. This allowed us to collect RT and ^DT^CCS_N2_ in parallel. ^DT^CCS_N2_ values from the single field experiment were in good agreement with values derived from the multifield method (Table 1). In order to identify potential trends for investigations in natural samples, we plotted the KMD for CH_2_ and RKMD against the *m/z*. As expected, homologous series form horizontal lines. Furthermore, the ^DT^CCS_N2_ was used as the size of data points (Fig. 2, see Supplementary Information (ESM) Table S1).

**Table 1:** Summary of CCS and RT values obtained maradolipid standards. CCS values were derived from direct infusion multifield measurements and UHPLC-IM-QToFMS. Means and standard deviations were calculated from triplicate measurements. Deviations of single field CCS from multifield CCS are indicated in brackets

Ion mode	Name	Adduct	m/z	^DT^CCS_N2_ ± SD in Å^2^ (stepped field)	^DT^CCS_N2_ ± SD in Å^2^ (single field)	RT ± SD in min
(+)	Mar(14:0/14:0)	[M+NH_4_]^+^	780.5467	282.87 ± 0.25	284.00 ± 0.26 (−0.40 %)	12.98 ± 0.03
	Mar(15:0/15:0)		808.578	289.13 ± 0.21	290.60 ± 0.20 (−0.51 %)	13.90 ± 0.02
	Mar(14:0/18:1)		834.5937	293.00 ± 0.20	294.20 ± 0.26 (−0.41 %)	14.38 ± 0.02
	Mar(16:0/16:0)		836.6093	294.93 ± 0.25	296.57 ± 0.38 (−0.55 %)	15.10 ± 0.02
	Mar(15:0/17:0)		836.6093	294.93 ± 0.25	296.63 ± 0.15 (−0.58 %)	15.47 ± 0.02
	Mar(15:0/18:1)		848.6093	296.03 ± 0.23	297.37 ± 0.35 (−0.45 %)	14.78 ± 0.02
	Mar(16:0/18:1)		862.625	298.77 ± 0.25	300.70 ± 0.26 (−0.65 %)	15.52 ± 0.02
	Mar(17:0/18:1)		876.6406	301.67 ± 0.15	303.17 ± 0.23 (−0.50 %)	15.85 ± 0.02
	Mar(18:1/18:1)		888.6406	302.87 ± 0.32	304.20 ± 0.00 (−0.44 %)	15.56 ± 0.02
	Mar(18:1/19:1)		902.6563	306.80 ± 0.17	307.67 ± 0.21 (−0.28 %)	16.23 ± 0.02
	Mar(14:0/14:0)	[M+Na]^+^	785.5021	---	282.70 ± 0.26	12.98 ± 0.02
	Mar(15:0/15:0)		813.5334	---	289.43 ± 0.32	13.90 ± 0.02
	Mar(14:0/18:1)		839.5491	---	291.47 ± 0.32	14.38 ± 0.02
	Mar(16:0/16:0)		841.5647	---	295.30 ± 0.36	15.09 ± 0.02
	Mar(15:0/17:0)		841.5647	---	295.37 ± 0.21	15.47 ± 0.02
	Mar(15:0/18:1)		853.5647	---	294.93 ± 0.31	14.78 ± 0.02
	Mar(16:0/18:1)		867.5804	---	298.83 ± 0.31	15.52 ± 0.02
	Mar(17:0/18:1)		881.596	---	301.73 ± 0.21	15.85 ± 0.02
	Mar(18:1/18:1)		893.596	---	303.33 ± 0.15	15.56 ± 0.02
	Mar(18:1/19:1)		907.6117	---	306.70 ± 0.20	16.23 ± 0.02
(−)	Mar(14:0/14:0)	[M+FA-H]^-^	807.5111	284.65 ± 0.35	284.40 ± 0.10 (−0.09 %)	12.94 ± 0.04
	Mar(15:0/15:0)		835.5424	290.55 ± 0.64	290.77 ± 0.06 (0.07 %)	13.87 ± 0.04
	Mar(14:0/18:1)		861.5581	293.95 ± 0.64	294.37 ± 0.40 (0.14 %)	14.35 ± 0.03
	Mar(16:0/16:0)		863.5737	296.35 ± 0.07	297.03 ± 0.51 (0.23 %)	15.06 ± 0.03
	Mar(15:0/17:0)		863.5737	296.35 ± 0.07	297.07 ± 0.38 (0.24 %)	15.44 ± 0.04
	Mar(15:0/18:1)		875.5737	297.20 ± 0.57	297.10 ± 0.53 (−0.03 %)	14.74 ± 0.04
	Mar(16:0/18:1)		889.5894	299.90 ± 0.28	300.07 ± 0.59 (0.06 %)	15.48 ± 0.04
	Mar(17:0/18:1)		903.605	301.85 ± 0.35	303.17 ± 0.46 (0.44 % )	15.82 ± 0.03
	Mar(18:1/18:1)		915.605	304.00 ± 0.42	303.13 ± 0.42 (−0.29 %)	15.53 ± 0.03
	Mar(18:1/19:1)		929.6207	306.70 ± 0.57	307.17 ± 0.40 (0.15 %)	16.20 ± 0..03

In contrast to glycerophospholipids, the maradolipids have no distinct sn1 or sn2 position since the 6 and 6′ position on the trehalose are equal. Therefore, only single peaks will be measured throughout the measurements, while for glycerophospholipids, two peaks might be found in the UHPLC and IM dimension. Similar to PCs or PEs, maradolipids show a linear increase in ^DT^CCS_N2_ with growing chain length. Slopes of trendlines for ^DT^CCS_N2_ vs *m/z* plots are slightly smaller for maradolipids compared with PCs and PEs (data not shown). In contrast to IM-MS alone, UHPLC-IM-Q-ToFMS was able to separate the isobaric structures Mar(16:0/16:0) and Mar(15:0/17:0) (Table 1).

Putative isomeric overlap within a 5mDa window in negative ion mode with theoretical PE-Cers and SMs with a high number of hydroxyl groups was found using the LipidMaps search against CompDB [21]. Since such lipids are currently not known in *C. elegans* and not expected, therefore collective information on the MS^1^ level (*m/z*, RKMD and ^DT^CCS_N2_) allow to identify putative maradolipid candidates in lipid extracts.

### Fragmentation pattern of maradolipids

Fragmentation patterns of maradolipid standards were investigated using UHPLC-IM-Q-ToFMS/MS with a 4 Da isolation window and targeted fragmentation. First, fragmentation in negative mode was investigated. Fragmentation pathways of acetate adducts of maradolipids have been described by Papan et al. [12]. Upon fragmentation, first the [M-H]^−^ ion is formed from which the fatty acids are lost and can be detected as free acyl or as neutral losses. Subsequently, fragments with *m/z* 323.0984 and 305.0878 derived from trehalose are formed.

Investigating the fragmentation of in our case [M+FA-H]^−^ adducts, similar fragmentation was observed. Fragmentation data of Mar(14:0/14:0) and Mar(14:0/18:1) was closer examined, both representing a symmetrical and an unsymmetrical maradolipid. Similar to the fragmentation observed by Papan et al., first the fragmentation of the [M+FA-H]^−^ to the [M-H]^−^ ion was observed. This fragment further fragments by losing one of the two possible fatty acids attached at the 6- or 6′-position which leads to [M-R_1_COOH]^−^ or [M-R_2_COOH]^−^ fragments. In case of Mar(14:0/14:0), only one single fragment and, in case of Mar(14:0/18:1), two fragments have been observed. The corresponding [R_1_COO]^−^ and [R_2_COO]^−^ fragments were also observed. The fragments [M-R_1_COOH]^−^ and [M-R_2_COOH]^−^ were only observed upon fragmentation with 20 eV. A total of 40 eV yielded highest intensities of [R_1_COO]^−^ and [R_2_COO]^−^. An interesting feature for the screening and putative identification for maradolipids are the fragments *m/z* 323.0984 and 305.0878 which correspond to [trehalose-H_2_O-H]^−^ and [trehalose-2 H_2_O-H]^−^.

**Fig. 3:**
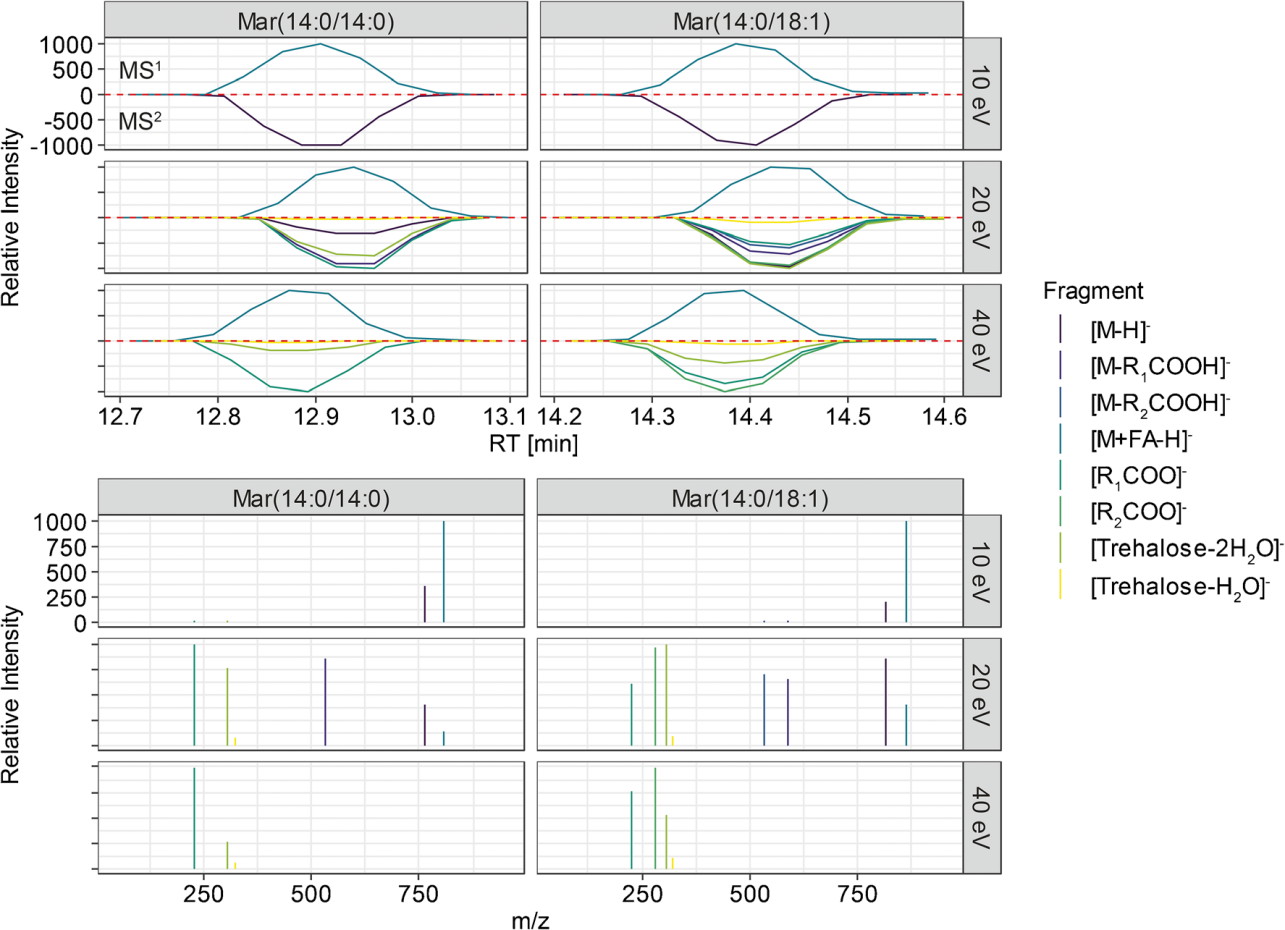
MS^2^ spectra are reconstructed using correlation of drift time filtered EICs. Different fragmentation energies yield different amount of fragments with different intensities. Candidate peaks with a high correlation coefficient as well as an explainable fragment m/z are used for identification. The plots show the EICs for Mar(14:0/14:0) and Mar(14:0/18:1) as example.

Data were collected using UHPLC-IM-Q-ToFMS and DIA fragmentation with alternating frames switching between low and high collision energy. Three different runs with either 10, 20, or 40 eV collision energy were produced. We aimed to investigate if UHPLC and IM-MS combined with DIA allows to obtain sufficient information for maradolipid identification. Co-elution and similarity in drift times allow to filter the DIA MS^2^ data and exclude false positive fragments. We therefore investigated for all maradolipid standards how elution profiles for fragments are behaving in comparison with the precursor. EICs for the respective fragment m/z and drift region were generated and correlated against the EIC of the precursor in the respective retention time region. We generally observed high correlation coefficients above 0.9 indicating that indeed the correct fragments are assigned. Figure 3 shows examples for the two standards Mar(14:0/14:0) and Mar(14:0/18:1). Reference spectra from negative ionization and targeted fragmentation are available in MassBank record format in the ESM.

Investigation of positive ion mode fragmentation data showed that major fragments derived from [M+NH_4_]^+^ adducts are [M-H_2_O+H]^+^ as well as [R_1_CO]^+^ and [R_2_CO]^+^ of the two respective acyl groups (data not shown). Since no additional information can be derived from combined positive and negative mode analysis, only negative mode data was further investigated.

Based on the obtained results, 20 eV seem to be the most informative collision energy, when performing non-targeted analysis and search for maradolipids since it yielded the most explainable fragments in a single collision energy. A total of 40 eV yielded the highest intensity for FA and trehalose fragments. Based on this result, we proposed to use UHPLC-IM-QToFMS with DIA and a collision energy 20 eV to screen for potential maradolipids in biological samples.

### UHPLC-IM-Q-ToFMS analysis of *C. elegans* dauer larvae

**Fig. 4:**
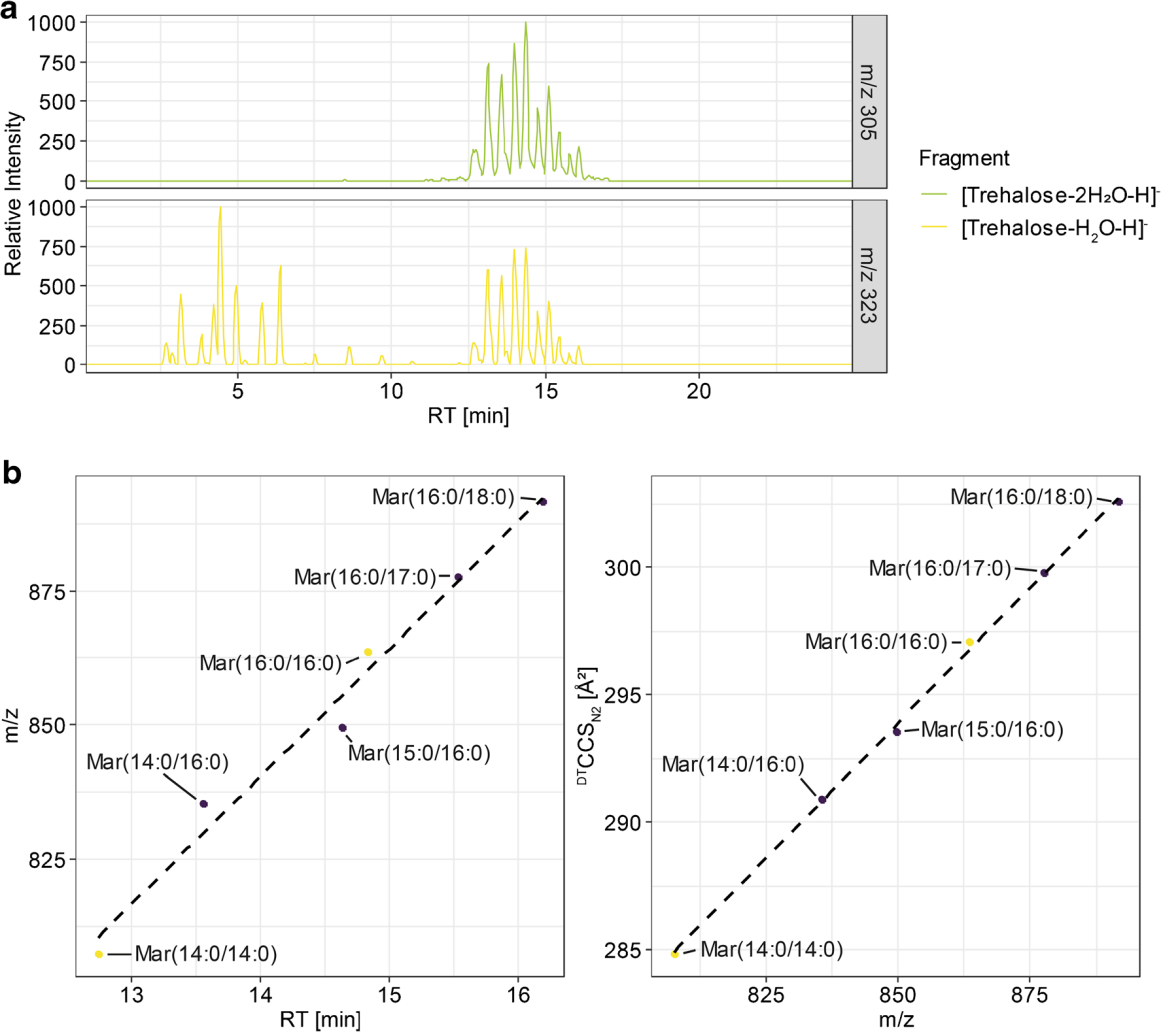
**a** Extracted ion chromatograms for m/z 305.0877 and 323.0972 in high collision energy frames of the UHPLC-IM-Q-ToFMS AllIons experiments. Coelution of both masses indicates presence of of maradolipids, while m/z 323.0972 alone indicates potential lysomaradolipids **b** RT and ^DT^CCS_N2_ trendlines used for identification of Mar(14:0/16:0). Yellow points represent reference standards, while all others are derived from maradolipids detected in dauer larvae extracts.

Our analysis of maradolipids using UHPLC-IM-Q-ToFMS showed that the combination of UHPLC, IM, and DIA can be used for the identification of maradolipids. In order to prove that this combination is able to identify maradolipids also in biological extracts, *C. elegans* dauer larvae were generated from *daf-2(e1370)* mutants by growing them at 25 °C. Worms were harvested and extracted using a Bligh and Dyer extraction. Analysis of dauer larvae was performed by UHPLC-IM-Q-ToFMS using DIA fragmentation with either 10, 20, and 40 eV. Since the positive mode did not offer additional information on the identification of maradolipids, only the negative mode data were used. To see first if maradolipids are found in the lipid extract, negative mode data were used, and extracted ion chromatograms for *m/z* 323.0972 and 305.0877 in the high collision energy frames were generated (Fig. 4a). Coelution of these two *m/z* indicates presence of potential maradolipids. Since 20 eV spectra contained the highest information content, they were investigated first. Indeed, coelution of the two m/z was observed in the range of 12 to 17 min, being in the same range where the standards are eluting. Interestingly, additional peaks for the *m/z* 323.0972 were observed in the range from 2.5 to 6.5 min, but not for *m/z* 305.0867.

Indicating the presence of potential maradolipids in the dauer extract, non-targeted peak picking of lipid features was performed. In total, 1349 features were detected in all three replicates of dauer larvae lipid extract in negative ion mode. From the measured m/z value, the KM, KMD, and RKMD were calculated according to Lerno et al. [22]. Using an error of ± 0.1 for the RKMD, the total list was narrowed down to 123 potential maradolipid candidates. The list was further condensed by filtering on the RT region of eluting maradolipid standards and compared against a computer-generated list of potential maradolipids using potential fatty acids present in maradolipids based on results from Penkov et al. (see ESM Table S4). Using MS^1^ annotation to filter potential maradolipids, 33 candidates remained. Of these, 10 could be matched with the used standards based on *m/z*, RT and ^DT^CCS_N2_ values as well as fragmentation pattern.

Investigating peaks that are putatively annotated as additional maradolipids, several interesting candidates were found. For example, *m/z* 835.5424 showed a small side peak in addition to the peak matched with the Mar(15:0/15:0) standard, which might represent an isobaric species with a different fatty acid composition. Investigating the DIA fragmentation data, it was putatively identified as Mar(14:0/16:0). To further confirm this putative identification, we checked trends along RT and ^DT^CCS_N2_ values. Data were checked for maradolipids that contained 14:0 and 16:0 fatty acyl side chains. Mar(14:0/14:0) and Mar(16:0/16:0) have been measured as standard. The putative Mar(14:0/16:0) falls between these standards in regard to RT and CCS (Fig. 4b). Although deviation of the Mar(16:0/16:0) standard from the RT trendline was higher, trends along ^DT^CCS_N2_ trend lines were fitting. Generally, a higher deviation of RT from standards was observed for maradolipids in *C. elegans* samples, but errors were generally below 2%, while the highest error for ^DT^CCS_N2_ was 0.4%. Furthermore, ^DT^CCS_N2_ trend lines showed good linear trends, while for RT, this was only the case for very limited examples and typically showed quadratic behavior. Combining all available information, the peak can be putatively to be Mar(14:0/16:0) based on DIA fragmentation data, RT and ^DT^CCS_N2_. Penkov et al. also detected Mar(14:0/16:0), and compared with Mar(15:0/15:0), it also showed lower levels. A list with all putatively identified maradolipids, their RT, *m/z*, and ^DT^CCS_N2_ values can be found in the ESM (see ESM Table S2).

### Lysomaradolipids

While searching for potential maradolipids using DIA fragmentation, an additional region between 2.5 and 6.5 min showing the fragment *m/z* 323.0972 was identified. However, no corresponding fragment *m/z* 305.0877 was found. Therefore, it was hypothesized that the peaks in this area might represent lysomaradolipids. Papan et al. have identified lysomaradolipids using shotgun-based lipidomics analysis of lipid extracts from *C. elegans* dauer larvae. The fragmentation pattern they have obtained shows strong similarities compared with the ones found in the present publication [12]. Their proposed fragmentation is matching the observation of the peaks eluting in this RT range. Using the obtained DIA fragmentation data, it was observed that a collision energy of 10 eV is more useful for the non-targeted search because the [M+FA-H]^−^ and [M-H]^−^ ions, as well as the [trehalose-H_2_O-H]^−^ fragments, are present in the high collision energy data. For further structural elucidation, 20 eV collision energy was used, since both the [trehalose-H_2_O-H]^−^ fragment as well as fatty acyl fragments were visible, while 40 eV mostly produced the fatty acyl fragment.

**Fig. 5:**
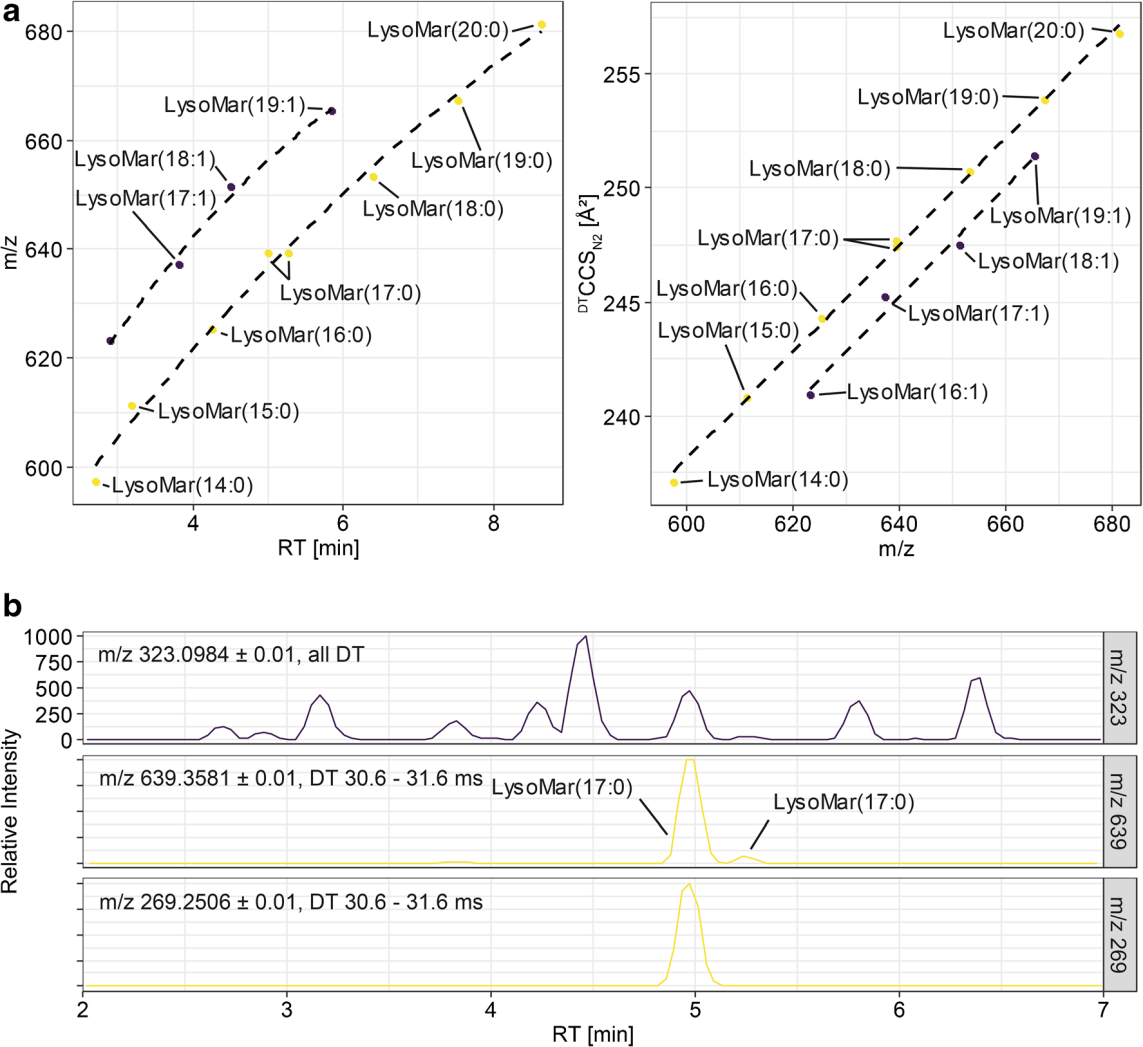
**a** RT and ^DT^CCS_N2_ trendlines constructed for lysomaradolipids. For LysoMar(17:0), two peaks are visible. Two trendlines have been constructed, one for saturated and one for mono-unsaturated lysomaradolipids. **b** Extracted ion chromatograms from low and high collision energy frames from AllIons fragmentation data. The two LyosMar(17:0) isomers were clearly separated in the RT dimension. Coelution for both peaks, the fragment m/z 323.0972 is observed. However, only for the first isomer, the respective FA(17:0) fragment was detected

Using a similar filtering approach and putative annotation on the MS^1^ level with masses of theoretical lysomaradolipids, we identified a list of 12 potential candidates. Coelution of MS^1^ *m/z*, the [trehalose-H_2_O-H]^−^ fragment as well as specific fatty acyl fragments were used for identification. Fragment *m/z* EICs were isolated for the specific drift time regions of the intact molecule and peak correlation was performed. Figure 5 a shows trendlines for lysomaradolipids.

Interestingly, for the *m/z* of LysoMar(17:0), two chromatographic peaks were found. While for the first and higher peak, fragmentation data identified a fragment at *m/z* 269, no fragmentation data confirming the putative ID was available for the second peak due to low intensity of the precursor. However, while checking for coelution with *m/z* 323.0972, perfect coelution could be observed for both peaks (Fig. 5b). *C. elegans* is able to produce mono-methyl-branched chain fatty acids on its own and most maradolipids contain a branched chain fatty acid. It might be possible that one peak represents a lysomaradolipid containing 15-methylpalmitic acid and the other one heptadecanoic acid. Both fatty acids have been detected in the analysis of total fatty acids, but heptadecanoic acid only in low amounts [23]. Investigating trendlines for both RT and CCS using odd numbered LysoMar showed that both peaks are matching the trends between LysoMar(15:0) and LysoMar(19:0). However, if only higher peak eluting earlier is used, trends increased. The ^DT^CCS_N2_ value of the second peak is slightly higher (247.72 Å^2^ compared with 247.44 Å^2^), which indicates a slightly larger structure. Since the two peaks showed good chromatographic separation, the logP values for both possibilities were calculated as a measure of hydrophobicity. The logP of the hypothetical straight chain LysoMar(17:0) is 2.66 and the logP of the hypothetical iso-branched chain version is 2.50. This would fit with the trends seen based on ^DT^CCS_N2_, indicating that the branched chain version is eluting before the straight chain version. However, these identifications are only putative and need to be confirmed with authentic standards. Table 2 summarizes all putatively identified lysomaradolipids (see also ESM Table S3). Since no reference standards are currently available for lysomaradolipids, these identifications cannot be further validated.

**Table 2:** Summary of ^DT^CCS_N2_ and RT values of detected lysomaradolipids. CCS values were derived UHPLC-IM-QToFMS. Means and standard deviations were calculated from triplicate measurements

Ion mode	Name	Adduct	m/z	^DT^CCS_N2_ ± SD in Å^2^ (multifield)	^DT^CCS_N2_ ± SD in Å^2^ (single field)	RT ± SD in min
neg	LysoMar(19:1)	[M+FA-H]−	665.3748	---	251.45 ± 0.35	5.839 ± 0.06
	LysoMar(16:1)		623.3281	---	240.94 ± 0.14	2.9 ± 0.01
	LysoMar(18:1)		651.3596	---	247.47 ± 0.36	4.506 ± 0.07
	LysoMar(17:1)		637.3444	---	245.22 ± 0.1	3.819 ± 0.01
	LysoMar(17:0)		639.3581	---	247.72 ± 0.41	5.26 ± 0.01
	LysoMar(20:0)		681.4054	---	256.82 ± 0.37	8.63 ± 0.01
	LysoMar(18:0)		653.3747	---	250.72 ± 0.43	6.407 ± 0.05
	LysoMar(16:0)		625.3436	---	244.3 ± 0.40	4.254 ± 0.03
	LysoMar(17:0)		639.3593	---	247.44 ± 0.27	4.995 ± 0.03
	LysoMar(19:0)		667.3906	---	253.90 ± 0.13	7.518 ± 0.00
	LysoMar(14:0)		597.3125	---	237.07 ± 0.45	2.697 ± 0.02
	LysoMar(15:0)		611.3282	---	240.83 ± 034	3.182 ± 0.02

## Conclusion

Lipid analysis and identification represent a delicate, but important task in lipidomics and lipid profiling. Besides, MS and MS/MS orthogonal information such as RT and ^DT^CCS_N2_ can be helpful in identifying members of homologous series or to clean up fragmentation patterns. We used DIA fragmentation to obtain further structural information. We described the analysis of maradolipids, a class of lipids found exclusively in the dauer stage of *C. elegans*, using UHPLC-IM-Q-ToFMS. Previous analysis of maradolipids used high-resolution shotgun lipidomics. In this work, lipid extracts from *C. elegans* dauer larvae were directly analyzed without prior prefractionation and enrichment of glycolipids. Based on authentic reference standards, ^DT^CCS_N2_ values using the stepped and single field methods could be determined. Furthermore, RT and ^DT^CCS_N2_ trendlines have been established. Combination of KMD, RKMD, RT, and ^DT^CCS_N2_ analysis as well as DIA fragmentation data allowed the identification of several members of the maradolipid family. In total, 33 maradolipids were putatively identified and 10 confirmed by authentic standards. Compared with the list from Penkov et al., most of our found maradolipids were also detected by them. Although in total, we only detect 33 compared with 59 maradolipids, we did not use any prefractionation, but measured the obtained lipid extracts directly reducing sample handling and potential error source. Furthermore, several lysomaradolipids for which no reference standards are currently available could be identified, including two putative isomers of LysoMar(17:0). It remains elusive to which extend maradolipids also contain isomers with straight- or branched chain fatty acyls. Chromatographic methods using higher shape selectivity, e.g., C30 columns, might be required [24].

The obtained results show how RT, ^DT^CCS_N2_, and DIA fragmentation can be combined for the identification of novel lipid species. The created methodology might be not only applicable to *C. elegans*, but also other organisms. Given the structural similarity of maradolipids to acyltrehaloses produced by *Mycobacterium tuberculosis* analysis of bacterial glycolipids represents an interesting future application area.

## Supplementary information

ESM 1 (XLSX 91 kb)

ESM 2 (TXT 67 kb)

## Data Availability

^DT^CCS_N2_ RT and m/z values of maradolipid standards as well as marado- and lysomaradolipids detected in *C. elegans* are summarized in Tables S1-S3 (see ESM). Masses, formulae, m/z, and fragment m/z of theoretical marado- and lysomaradolipids are summarized in ESM Table S4. Reference MS^2^ spectra from maradolipid standards in MassBank format are available in ESM 2.
